# The evolution of cost-efficiency in neural networks during recovery from traumatic brain injury

**DOI:** 10.1371/journal.pone.0170541

**Published:** 2017-04-19

**Authors:** Arnab Roy, Rachel A. Bernier, Jianli Wang, Monica Benson, Jerry J. French, David C. Good, Frank G. Hillary

**Affiliations:** 1 Department of Psychology, The Pennsylvania State University, University Park, Pennsylvania, United States of America; 2 Department of Radiology, Hershey Medical Center, Hershey, Pennsylvania, United States of America; 3 Department of Neurology, Hershey Medical Center, Hershey, Pennsylvania, United States of America; 4 Social, Life and Engineering Sciences Imaging Center, University Park, Pennsylvania, United States of America; University of Cambridge, UNITED KINGDOM

## Abstract

A somewhat perplexing finding in the systems neuroscience has been the observation that physical injury to neural systems may result in enhanced functional connectivity (i.e., hyperconnectivity) relative to the typical network response. The consequences of local or global enhancement of functional connectivity remain uncertain and this is particularly true for the overall metabolic cost of the network. We examine the hyperconnectivity hypothesis in a sample of 14 individuals with TBI with data collected at approximately 3, 6, and 12 months following moderate and severe TBI. As anticipated, individuals with TBI showed increased network strength and cost early after injury, but by one-year post injury hyperconnectivity was more circumscribed to frontal DMN and temporal-parietal attentional control regions. Cost in these subregions was a significant predictor of cognitive performance. Cost-efficiency analysis in the Power 264 data parcellation suggested that at 6 months post injury the network requires higher cost connections to achieve high efficiency as compared to the network 12 months post injury. These results demonstrate that networks self-organize to re-establish connectivity while balancing cost-efficiency trade-offs.

## 1. Introduction

Each year in the United States there are approximately 1.5 million to 2 million cases of traumatic brain injury (TBI) resulting from an external source and causing significant physical, cognitive, and psychosocial impairment [1]. To aid in understanding the behavioral deficits associated with TBI, over the last decade, blood oxygen level dependent functional magnetic resonance imaging (BOLD-fMRI, or fMRI) has been widely used to study functional connectivity changes associated with injury. In this literature functional connectivity strength has commonly been defined as the degree of temporal-correlation in the fMRI signals recorded from two identified regions in the brain [2]. Brain connectivity studies have provided novel insights into the influence of TBI on neural networks during motor learning (see [3]), working memory and attentional control [4–6] and the relationship between distinct subnetworks and cognitive performance (e.g., default mode network, DMN and salience network, SN) [4,7–10]. Here our goal is to use BOLD-fMRI to advance this literature in three important ways. First we examine the phenomenon of functional hyperconnectivity (discussed below) with focus on the timing when it occurs during the first year of recovery after TBI, and the regions where it is most likely to be expressed. The second goal was to examine the inter-relationship between network cost and its achieved efficiency (or cost-efficiency relationship) during the recovery period. Finally, we examine hyperconnectivity in the context of cognitive performance. In doing so this study represents an opportunity to examine global/local neural network characteristics at three time points during the first year following moderate and severe TBI.

### 1.1 Functional hyperconnectivity

While much work using connectivity modeling in neurological disorders has focused on specific relationships between localized neuropathology and loss of functional connectivity (see [11–13] and for review see [14]) one commonly observed response to injury and disease is enhanced functional connectivity (henceforth referred to as hyperconnectivity). For the purposes of this paper, we will define hyperconnectivity as the increase in the strength of functional connections in the brain after injury relative to a healthy control (HC) sample. Given that the pathophysiology observed in moderate and severe TBI results in disruption of neural signaling, functional hyperconnectivity is a counterintuitive neural network response. In order to understand the nature of hyperconnectivity after TBI, we aim to examine distributed networks and document the evolution of connectivity changes during recovery with specific interest in the relationship between hyperconnectivity after TBI and *network cost* that remains unexplored.

### 1.2 Network cost and efficiency

Human brain functioning is the result of a finely tuned orchestra of billions of neurons oscillating across different spatio-temporal scales to achieve communication (i.e., connectivity). The functional connections achieved during states of signal transfer in a healthy brain are embedded within networks tuned to achieve optimal balance between network cost and routing efficiency [15–18]. We anticipate that the functional changes evident post injury and, in particular hyperconnectivity, may disrupt this optimal balance between the cost and efficiency. If it is the case that neurological disruption results in enhanced connectivity either in the magnitude or number of connections, even in select regions within the brain, then this should have important implications for overall network cost. The cost of the functional network can be defined using the total number or the weight of the connections in the functional network. This traditional way of defining network cost has one important drawback: it fails to consider the physical length of a functional-connection which has important implications for the latencies of neural transmission [19–20] and for the metabolic cost of signaling [21].

To capture network cost after injury in the current study and its consequences for efficiency, we include several novel metrics: for cost we summarize a) whole-brain network cost, and b) ROI-cost (see Section 2.6.3) and for cost-efficiency, we examine changes in global efficiency as a function of changes in cost. The cost metric is based upon three important properties of the functional network: the number of functional connections, the strength of each functional connection, and the physical length of each connection and this can be computed locally and globally. The goal is to determine if the relationship between cost and efficiency guides the evolution of neural network functioning during recovery from TBI. With the combined examination of connectivity strength and cost during a critical early window after moderate and severe TBI, the current study will advance our understanding of early network plasticity and its consequences for behavioral recovery.

### 1.3 Study goals and hypotheses

Our primary objective is to understand the early network response during the first year of recovery from moderate to severe TBI with specific focus on hyperconnectivity and negotiation of network cost and efficiency. In doing so, this will be the first study in TBI to examine neural network changes at three time points during the first year after injury. The primary hypothesis is that hyperconnectivity, defined as an increase in the number or strength of network connections, will be most evident during the early recovery period after TBI. With notable exceptions (see [22–23]) prior findings that have reported enhanced connectivity within the default mode network (DMN) and the executive control network (ECN) after moderate to severe TBI [3,6–7,10, 24–25], we hypothesize that hub regions within the DMN and ECN will primarily contribute to hyperconnectivity. Finally, we anticipate that network cost will be greater as a result of hyperconnectivity, but that networks are obliged to equilibrate cost-efficiency demands during recovery, so systems should gravitate to reduce network cost. As a result, we hypothesize that at one year post injury the functional network will achieve higher efficiency at a lower cost compared to the network early during the recovery period.

## 2. Materials and methods

### 2.1 Subjects

Subjects included 14 individuals who sustained moderate to severe traumatic brain injury between the ages of 18 and 36 with education ranging from 12 to 18 years, and 12 healthy controls of comparable age and education (see Table 1). A Glasgow Coma Scale (GCS) of 3–8 at time of injury was indicative of severe injury and a GCS of 9–12 was indicative of moderate injury [26]. Two subjects who originally received a GCS of 14 and one subject who received a GCS of 15 were also included because their medical records indicated positive imaging findings of brain damage and/or mental status change during their acute inpatient stay. Patients were excluded if there were other concomitant injuries (e.g. orthopedic injuries or injury to the spinal cord) that would cause difficulty for the patients to stay still in the MRI environment. All subjects with TBI completed three separate sessions approximately 3, 6, and 12 months following injury, referred to as Time-1, Time-2, and Time-3. In order to determine the natural variation in network response over two measurements separated by time, we also collected MRI data for healthy controls from two MRI sessions separated by approximately 3 months (see Table 1). Each testing session included both MRI data collection and cognitive testing. The goal in the current study is to extend hyperconnectivity hypotheses from our previous work to examine metrics of cost and efficiency using whole-brain graph theoretical analysis and to include an additional time-point during the first year after injury. To provide context with regard to other published work from our laboratory examining longitudinal recovery in TBI, the data used in this study maintain partial overlap with Venkatesan et al. (9/14 TBI subjects [10]). However, the analysis in Venkatesan et al., was a focused seed-based analysis of the PCC and hippocampus at two time points. The current data do not overlap with recent longitudinal work [8] which made use of task-based fMRI data also only at two time points post injury. Moreover, the current fMRI data sets are completely distinct from other prior work examining task-based connectivity [24, 25] and the samples in those studies maintain either no overlap [25] or minimal overlap (2/14 TBI subjects [24]) with the current sample.

**Table 1:** Subject demographics and TBI injury profiles.

**a. Demographics**	**Mean HC (sd)**	**Mean TBI (sd)**
Age	32.00 (11.5)	26.07 (6.5)
Education	12.92 (1.73)	13.42 (2.43)
Gender	6 M; 6 F	7 M; 7 F
GCS	N/A	7.92 (4.9)
Mos. post Time-1	N/A	3.36 (0.74)
Mos. post Time-2	N/A	6.64 (1.45)
**b. Mechanisms of injury**		**No. TBI (%)**
Motor Vehicle Accident		7 (50)
Fall		4 (29)
Struck by Vehicle		3 (21)
**c. Initial clinical CT/MRI findings**		**No. TBI (%)**
Diffuse Axonal Injury		7 (50)
Total Hematoma and/or Hemorrhage *		13 (93)
*HH Left Lateralized*		*5 (36)*
*HH Right Lateralized*		*3 (21)*
*HH Bilateral*		*4 (29)*
*HH Frontal Lobe*		*12 (86*
*HH Temporal Lobe*		*6 (43)*
*HH Parietal Lobe*		*4 (29)*
*HH Occipital Lobe*		*2 (14)*

GCS, Glasgow Coma Scale; Mos., months; No. TBI, number of TBI subjects; HH, Hematoma and/or Hemorrhage.

* One of the thirteen subjects with reports of intracranial hematoma and/or hemorrhage did not have a documented location of this finding

All conducted research was approved by the institutional review board and the Pennsylvania State University Office of Research Protections. Due to the nature of the study sample, some individuals included in the study demonstrated some level of cognitive impairment. However, all individuals in the study retained the ability to sign medical documents and/or functioned independently, so consent was accepted and caregiver consent was included when available.

### 2.2 Behavioral data

Each subject was administered a neuropsychological battery of tests at Time-1, and all TBI subjects were administered the same battery during subsequent time points. The battery was designed to examine cognitive domains associated with deficits in TBI, focusing on the domains of attention and working memory [27]. In order for the TBI sample to be capable of reliably completing this battery at 3-months post injury, the battery was focused on those tests most sensitive to deficits following TBI, including processing speed and working memory. Tests administered included the Visual Search and Attention Test (VSAT), WAIS-III Digit Span (Forward, Digits-F and Backward, Digits-B), Trail Making Test B (TMT), and Stroop Color and Color-Word (Stroop). Because normative data were not available for all tests (e.g., digit span forward v. backward, WAIS-III) mean and standard deviation of the TBI subjects’ raw scores were used during analyses. Independent samples t-test comparing mean performance at Time-1 for TBI with mean performance at time for HCs showed no differences in performance between groups. (see Table 2).

**Table 2:** Neuropsychological performance of TBI group.

	**Stroop mean (sd)**				**VSAT mean (sd)**
	**Color Time**	**Color Total**	**Color-Word Time**	**Color-word Total**	**Total**
**Time-1**	62.15 (10.56)	111.69 (0.63)	105.62 (23.01)	106.83 (10.15)	110.08 (21.89)
**Time-2**	67.58 (16.64)	111.75 (0.62)	109.92 (11.44)	96.25 (25.69)	121.77 (25.90)
**Time-3**	61.23 (9.88)	111.62 (0.87)	109.25 (11.68)	102.42 (19.85)	122.46 (19.03)
	**Digits: mean (sd)**			**Trails: mean (sd)**	
	**Forward**	**Back**	**Total**	**A**	**B**
**Time-1**	10.69 (2.02)	6.92 (2.02)	17.62 (3.40)	29.31 (11.32)	61.38 (16.91)
**Time-2**	10.67 (1.96)	7.92 (1.75)	18.69 (3.35)	25.08 (5.99)	49.31 (14.26)
**Time-3**	10.84 (1,59)	7.23 (1.79)	18.08 (2.69)	21.23 (4.21)	46.08 (10.17)

Stroop C, color trial; Stroop CW, color/word interference trial; VSAT, visual search and attention task.

### 2.3 Functional data

**S**ubjects were scanned using a Philips Achieva 3T scanner in the Department of Radiology at Hershey Medical Center, Hershey, PA (TBI n = 1; HC n = 6) or one of two identical Siemens Magnetom Trio 3T scanners (one at the Social, Life, and Engineering Sciences Imaging Center at the Pennsylvania State University in University Park, PA (TBI n = 1, HC = 4); the other at the Department of Radiology at Hershey Medical Center in Hershey, PA, (TBI n = 12; HC n = 2). TBI cases and HC cases were divided between the scanners and for repeated scanning, all subjects were scanned on the same machine across time points. Prior to scanning subjects were made aware of the importance of minimizing motion within the scanner. High resolution T_1_-weighted anatomical images of the brain were acquired with isotropic spatial resolution of 1.0 mm. Echo planar imaging (EPI) was used to examine the blood oxygen level dependent response for functioning imaging. Imaging parameters for EPI were 2000 ms/30 ms/90° (repetition time/echo time/flip angle), 240 × 240 mm^2^ field of view, and 80 × 80 acquisition matrix with 4 mm-thick axial slices parallel to the bicommissural line. This study demanded data collection over seven years using more than 1 MRI scanner, so formal analysis of differences in the temporal signal-to-noise-ratio (tSNR) was conducted to examine the possible contribution of voxelwise differences in SNR on the findings (see Supplemental Analysis of Scanner tSNR below).

### 2.4 fMRI data preprocessing

Resting data were collected over the course of a 5-minute period, resulting in 150 volumes of data. The first five volumes were removed from analyses to control for signal instability, resulting in a time series of 145 volumes. All preprocessing steps were conducted using SPM8 (http://www.fil.ion.ucl.ac.uk/spm/software/spm8/). For all volumes bad slices were first repaired using art-slice procedure, which is part of ArtRepair toolbox [28]. The volumes were then slice-timing corrected and realigned. Spike artifacts were eliminated using despike filter available in ArtRepair toolbox. For each subject their high-resolution T_1_-weighted anatomical image was co-registered to the mean functional image using SPM8. The co-registered anatomical image was then segmented using SPM8, which produced a normalized gray matter image in the standard Montreal Neurological Institute (MNI) brain space. The functional images were then normalized to the MNI space. The normalized functional volumes and the gray matter image were resliced to a spatial resolution of 3 mm isotropic. The equivalence of voxel size allowed one to one mapping of voxels between normalized functional and anatomical volumes, and provided a mechanism to extract gray matter signal (see the following section). To reduce the effect of ringing artifact and to improve signal to noise ratio [29], a 6-mm isotropic smoothing filter was applied to the normalized functional data. The cerebrospinal fluid (CSF) and the white matter nuisance signals were regressed out, and a bandpass filter of 0.01 Hz to 0.12 Hz was applied using CONN toolbox. Finally, all functional data were movement corrected using ArtRepair. The movement parameters have been summarized in Table 3.

**Table 3:** Motion correction summary.

	Mean PVR (sd)	Mean SD of data (sd)	Mean CT (sd)	Mean CT, % of mean (sd)
**TBI Time-1**	3.30 (6.49)	1.59 (0.58)	6.23 (2.48)	1.30 (0.01)
**TBI Time-2**	3.17 (6.87)	1.68 (1.02)	6.45 (3.12)	1.30 (0.02)
**TBI Time-3**	3.05 (4.72)	1.67 (0.61)	5.82 (1.89)	1.30 (0.01)
**HC Time-1**	2.47 (2.70)	1.76 (0.88)	6.30 (1.91)	1.30 (0.01)
**HC Time-2**	0.86 (2.14)	1.67 (0.57)	6.10 (1.67)	1.30 (0.01)

PVR, percent of volumes repaired; SD, standard deviation; CT, current threshold.

#### 2.4.1 Head motion correction

The removal of nuisance signal due to physiology and head motion is a developing issue receiving significant recent attention in the resting MRI literature [30,31]. To correct motion, raw data were examined for motion- related slice and volume signal changes using ArtRepair [28] and standard 6-parameter realignment to serve as null regressors. Using this pipeline reveals that in our TBI Time-1 group, where motion is typically the greatest concern, we can resolve motion problems in ~89% of the scans using standard ArtRepair guidelines (25% volume correction; exclusion of 7 data sets of 63 total (21 subjects originally enrolled, 3 time points each). Power and colleagues (2012) demonstrate that global signal regression is a powerful method for reducing the effects of motion. Given concerns published elsewhere [32,33] we did not employ that approach. Results of motion correction are available in Table 3.

### 2.5 Volumetric values

To help constrain the interpretation of neural network connectivity we integrate information about brain volume change over time. The relative volumes of white matter, and gray matter relative to the intracranial volume were calculated for each subject at each time point using the VBM library in SPM8. To detect if there was significant brain atrophy after the injury, the volumes of gray and white matters of later time points were compared with Time 1 (Paired t-test, with multiple comparison correction for the TBI group) (See Table 4).

**Table 4:** VBM analysis: Volume ratio.

	Mean GM/T (sd)	Mean WM/T (sd)
**TBI Time-1**	0.471 (0.020)	0.370 (0.014)*
**TBI Time-2**	0.472 (0.017)	0.364 (0.017)
**TBI Time-3**	0.481 (0.013)	0.357 (0.023)*
**HC Time-1**	0.485 (0.025)	0.354 (0.017)
**HC Time-2**	0.483 (0.026)	0.353 (0.017)

GM/T, gray matter volume / total volume; WM/T, white matter volume / total volume.

* *p* = 0.024 calculated by pairwise t-tests

#### 2.5.1 Lesion volumetrics

TBI is highly heterogeneous with respect to injury severity and pathophysiology. To address this issue and determine the influence of global pathology on brain networks, we created a 3-dimensional (3D) lesion model for each subject using 3D Slicer [34] that utilizes information from multiple MR sequences, such as SWI, FLAIR, and T1 MPRAGE. In the first step of the lesion modelling process, a neuroradiologist manually located and labelled all the hemorrhage and edema lesions in the three image modalities from each subject. During the lesion modelling process, first the multimodality MR images (SWI, FLAIR, and T1 MPRAGE) were co-registered, and then the lesions were segmented semi-automatically with the Atlas Based Classification (ABC) toolkit based on the labelling in the previous step. Finally, a 3D rendering of all lesions was generated and verified by the same neuroradiologist. As the goal here was to examine the overall effect of lesions on the global network metrics, we merged both lesion types (edema and hemorrhage) and lesion volume was calculated as the ratio of lesion volume to gray matter volume.

### 2.6 Analytic approach and pipeline

To test our hypotheses (See section 1.2), a functional connectivity network for each subject at each time-point was developed, and a subject-specific connectivity profile was created using the functional network. The connectivity profile included metrics that summarize total functional connections, network strength, network cost, and network efficiency.

In this article we performed two types of analyses: global and local analysis. For both these analyses the following computational steps were implemented: 1. A subject-specific binary gray matter mask at each time point was developed; 2. ROIs were defined using Power’s 264 anatomical coordinates and for each subject at each time point using the mean signal of each ROI to evaluate functional connectivity. 3. Using graph theoretical procedures a connectivity profile was developed for each subject at each time point.

In global analysis, we focused on examining the whole-brain connectivity changes during recovery period and to determine if these changes predict behavior. Finally, we also performed lesion analysis to determine the influence of focal pathology on global network metrics. In order to guarantee that our approach to brain parcellation was not a primary determinant of the regional and global network effects, we conducted a second brain parcellation using spatially constrained independent components analysis (sICA) method (see [35]) followed by a graph theoretical analysis for comparison (see Supplementary Analyses below).

**Fig 1:**
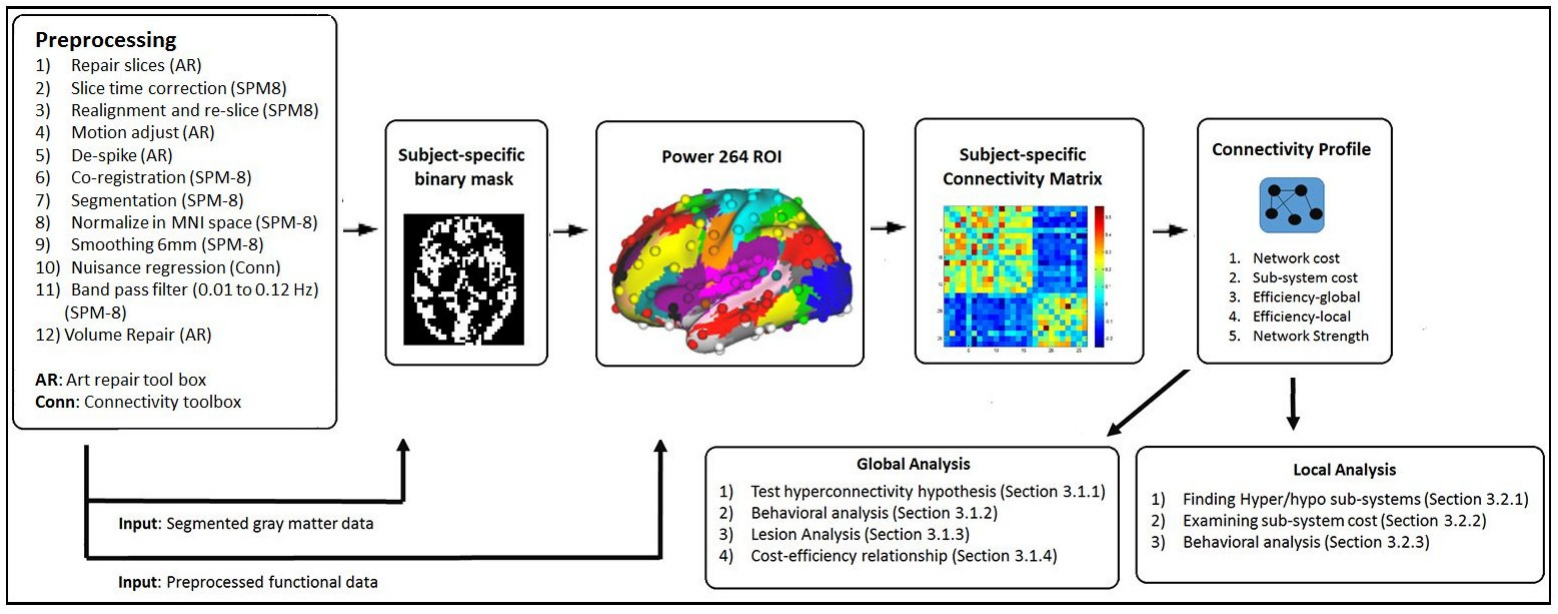
fMRI data preprocessing steps and the analytic pipeline.

In local analysis, we focus on finding regions (i.e. subsystems) in the brain that are consistently hyper- or hypo-connected at all 3 time points and also examine their cost. Further analysis using these hyper- hypo-connected subsystems are then conducted for only the TBI group. The analytic pipeline along with the preprocessing steps are illustrated in Fig 1. These steps are discussed in a greater detail in the following sub-sections.

#### 2.6.1. Subject-specific binary gray matter mask

A specific binary mask for each subject at each time point was created to better capture the change in the volumetric values across time. The segmentation procedure in SPM8 assigns a probabilistic value to each voxel of the gray matter segmented file (i.e. the probability that the voxel belongs to the gray matter). Therefore, to develop a binary gray matter mask, we had to use a threshold. We used those voxels with probability for gray matter greater than 0.6 as suggested by Zalesky and colleagues [36]. For each subject their binary gray matter mask was then overlaid on the normalized functional images to extract the BOLD signal from the gray matter voxels.

#### 2.6.2. Developing subject-specific functional connectivity network

We used Power's 264 anatomical coordinates [37] to segment the brain into 264 regions of interests (ROIs). ROIs were defined as 15 mm spheres centered at these coordinates. Power's 264 anatomical coordinates represent putative functional areas of the brain, and therefore, this way of defining ROIs provides a mechanism to locate specific subnetworks as well as anatomic landmarks in the brain that are most affected during recovery from TBI.

For each subject the average signal time course of the gray matter voxels in each ROI was measured, and Pearson's correlation between the signal time courses of all possible pairs of ROIs was conducted. The correlations that fail the false discovery rate (FDR) at a *p*-threshold of 0.05 were discarded. The remaining set of correlation values thus represent a subject specific weighted functional connectivity network.

#### 2.6.3 Subject-specific connectivity profile

The following network metrics were evaluated: 1) Total functional connections, 2) Network strength, 3) Local efficiency: clustering coefficient, 4) Global efficiency: average shortest path length, 5) Network cost.

The total functional connectivity in the brain for each subject at each time point was evaluated by counting the total number of statistically significant (FDR criterion) functional edges. This metric was used to determine the relative network connectivity.

Network strength was defined as the total sum of the absolute values of the network weights. The correlation coefficient between each pair of ROIs represents the weight of the connection. The rationale behind using the absolute value was to be able to examine the magnitude of the synchronization between ROIs. If the magnitude of s synchronization is high, it may be indicative of high metabolic consumption [38]. Additionally, we defined ROI-strength as the total strength of all functional edges that are incident upon a ROI. This allowed us to isolate brain subsystems that showed hyperconnectivity.

In order to examine local communication efficiency within the network, we calculated local clustering coefficient. Local clustering coefficient of vertex *i* is defined as:

$$C_{i}=\frac{\left|\left\{e_{jk}:v_{j},v_{k}\in N_{i},e_{jk}\in E\right\}\right|}{k_{i}\left(k_{i}-1\right)}$$ (1)

where *k*_*i*_ is total number of neighbors (i.e. neighboring nodes) of node *i*, *N*_*i*_ represents the set of all nodes that are neighbors of node *i*, *v*_*j*_ and *v*_*k*_ are vertices in the neighborhood of *i; E* is the set of all edges in the network, *and e*_*jk*_ represents the connections that exist between the neighbors of node *i*. A high clustering coefficient would mean that the neighbors of node *i* are well connected, hence, suggesting a high local efficiency around node *i*.

In order to examine global network efficiency, shortest path length, *d(i*,*j)*, is computed between all pairs of nodes *i* and *j*. The global efficiency is then defined as follows:

$$E\left(G\right)=\frac{2}{n\left(n-1\right)}\sum_{i<j\in G}\frac{1}{d\left(i,j\right)}$$ (2)

A measure of network cost was derived based on the assumption that the synchronization between long distance ROIs must be more metabolically expensive than the short distance ROIs. We defined the cost of each functional edge as the product of the Euclidean distance between the ROI-pair and the absolute weight of the connection. The overall network cost was then derived by totaling the cost of all edges that are part of the network. This definition of the network cost encapsulates 3 different concepts into one: level of synchronization between all pair of ROIs (network strength), number of functional edges, and physical distance of the functional edges.

Additionally, we defined ROI-cost as the total cost of all functional edges that are incident upon a ROI. This allowed us to isolate brain subsystems that make more expensive connections in an injured brain comparing to the HCs.

#### 2.6.4 Global and local analysis

Once the subject specific connectivity profiles are developed, we perform the following global and local analyses to test the study hypotheses (see Section 1.3). First, a global analysis was performed comparing network strength between the TBI and HC samples; the comparison is performed at all three time points (TBI Time vs. avg HC). To determine if differences in strength predicts cost, we also conducted a between group comparison of global network cost. We then examine how the cost-efficiency relationships change during the recovery period. This is accomplished by performing simulations to test network efficiency for different cost values at all three time points. We then examined the relationship between global network metrics and cognitive functioning. Finally, we examine the relationship between brain lesions for all TBI cases at Time-1 network connectivity and behavior.

For local analysis, we examined the subregions that contribute to hyperconnectivity, we mapped the Power-ROIs to 20 functionally defined subsystems. These 20 sub-networks were based upon prior findings in the TBI the literature and focused on the brain’s most robust sub-networks. While one goal of the study was to focus on within TBI group change during the first year, we first isolate the most high-strength and high-cost sub-regions in TBIs comparing to the HCs. For these isolated subsystems, we calculate the connection cost of ROIs that are part of these subsystems, and then analyze connection metrics (connection count, strength, and physical distance) that govern the change in their cost across time. Finally, we examine the relationship between the overall cost of these subsystems and behavioral performances.

#### 2.6.5 Supplementary analyses

Implementing an identical pre-processing pipeline as above, we substituted a spatially constrained ICA as the brain parcellation method to determine if brain parcellation was an important determinant of the network findings. There are, of course, important limitations to these types of comparisons (see [39]) so the goal was not to achieve identical results when comparing networks of distinct sizes. Instead the primary goal was to determine if the hubs driving cost-efficiency tradeoffs were commensurate. We chose ICA as a second approach for two reasons. First, in contrast to Power-264 based parcellation scheme where the representative signal for a ROI is developed by averaging over all voxels with in the ROI, in ICA, the representative signal is established using a weighted average; the ICA procedure estimates a weight for each voxel using an adaptive algorithm. This provides us an opportunity to examine if the parcellation scheme and also the mechanism by which ROI representative signals are developed affects the results. Second, ICA is a well-established approach receiving widespread application across a number of clinical samples (for review see [40–41]). While details for this analysis are consistent with [8], in brief, the procedure included the Group ICA of fMRI Toolbox (GIFT). To achieve a detailed component structure and because a higher ordered dataset was desirable for graph theoretical analyses, we chose a relatively high model order ICA (100 components) for all analyses [35, 42–44]. Preprocessing included movement correction, coregistration, and normalization, but consistent with [35] we did not remove signal from cerebrospinal fluid and white matter and did not conduct volumewise correction prior to the ICA. Subject-specific data reduction principal components analysis retained 120 principal components and group data retained 100 principal components followed by a component sort based upon dynamic range and low/high frequency ratio using 0.01 and 0.1 Hz as guidelines (consistent with [35]). The time series from retained components were submitted to an adjacency matrix and connections were FDR corrected resulting in an edge list that was submitted to graph theoretical analysis.

## 3. Results

### 3.1 Global analysis

#### 3.1.1 Examining hyperconnectivity

To examine the hyperconnectivity hypothesis, we used analysis of covariance (ANCOVA) with MRI scanner as a covariate in order to compare the TBI distributions for network strength at each time-point to the mean HC distribution of network strength; the mean HC distribution was established by averaging the 2 time-point HC distributions. As expected, effects at the global level were subtle; heightened connectivity at a global scale for network strength (p = 0.049; η_p_^2^ = 0.16) and number of links (p = 0.046; η_p_^2^ = 0.16) was observable only at time-2 (p = 0.049; η_p_^2^ = 0.16).

We also performed a similar analysis for the global network metrics, including clustering coefficient and mean path length, to determine how network efficiency may be affected due to TBI; the analysis revealed subtle effects for path length and clustering coefficient at Time-2 (see Table 5). A more nuanced analysis of cost and efficiency is performed in the following sub-section (see Section 3.1.4).

**Table 5:** Summary of graph network metrics (mean; sd).

	Avg Short PL-U	Avg Local CC-U	Avg Local CC-W	Network Strength	Average Number Links
**TBI Time-1**	1.70 (0.07)	0.40 (0.08)	0.42 (0.08)	3363.56 (953.84)	10504.64 (2528.39)
**TBI Time-2**	1.69 (0.05)*	0.41 (0.05)*	0.43 (0.05)*	3456.77 (682.07)*	10822 (1886.06)*
**TBI Time-3**	1.71 (0.04)	0.39 (0.04)	0.41 (0.04)	3171.87 (509.80)	9988.85 (1418.27)
**HC Average**	1.71 (0.02)	0.38 (0.02)	0.40 (0.02)	3093.64 (284.43)	9784.29 (819.68)

Avg Short PL-U: Average shortest path length (unweighted); Avg Local CC-U: Average local clustering coefficient (unweighted); Avg Local CC-W: Average local clustering coefficient (weighted); AFC.

* *p* < 0.05 calculated by ANCOVA, with MRI scanner as a covariate.

#### 3.1.2 Lesion analysis

**Fig 2:**
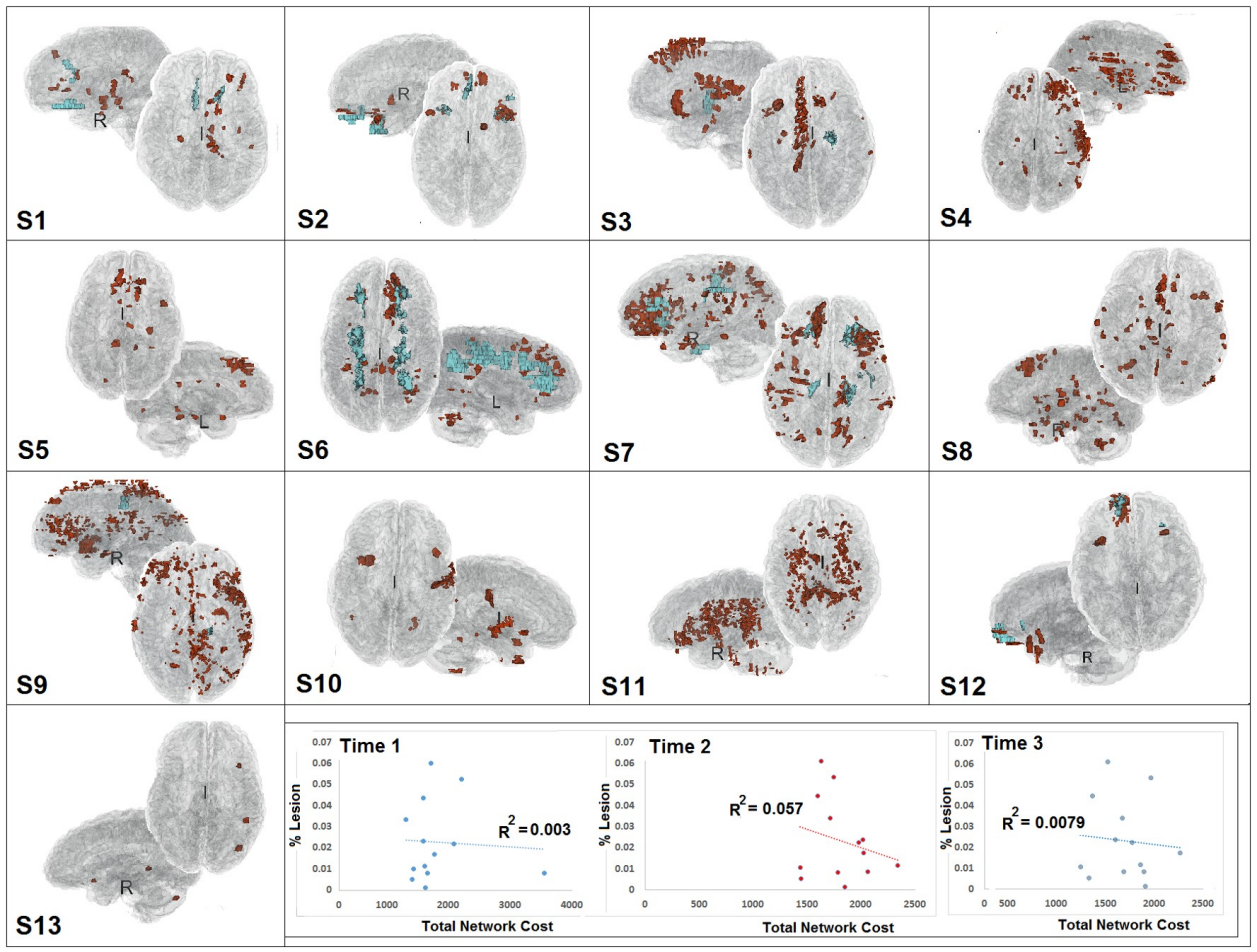
Results of 3D lesion modeling for TBI subjects at Time 1 (approximately 3-months post injury). Total network cost and the lesion volume are not significantly correlated at Time 1, Time 2, or Time 3.

The results of the 3D rendering procedure produced identifiable lesions in 13/14 cases (Fig 2). In the case showing no conspicuous hemorrhage or contusion, we anticipate that the injury was milder and/or diffuse. Results of the lesion analysis of the 13 cases with identifiable trauma lesions is illustrated in Fig 2. To examine the influence of total lesion, we collapsed for hemorrhage and contusion and calculated a percentage of lesion volume to total gray matter volume at the individual subject level. Unsurprisingly, the results do not show a significant relationship between lesion status and cognitive functioning based upon composite scores (*r-values ranging from r =* -.02 to -0.22). This finding is consistent with a TBI literature revealing inconsistent relationships between structural brain changes and cognitive outcome after TBI [45–48]. Proportion of lesion volume also did not predict unweighted path length in the network (T1 r-value = 0.01, T2 r = 0.20, and T3 r = 0.01) and did not predict global network cost (T1 r = 0.05; T2 r = 0.24; T3 r = 0.09). Overall, the 3D lesion rendering provided some context for these injuries, but was not significantly predictive of patient outcome or the network response.

#### 3.1.3 Examining the cost-efficiency relationship

We aimed to examine cost-efficiency relationship over time with focus on Time-2 and Time-3 as high efficiency/high strength was observed at Time-2 but not at Time-3 (based upon Table 5). High “efficiency” at Time-2 is a secondary effect of its cost: increasing the number of links results in lower path length, but the question remains whether Time-2 maintains superior cost-efficiency organization in the absence of these additional links. The approach taken was to randomly delete edges to test the cost-efficiency of the network as it becomes increasingly sparse. As an analogue, investigators have used similar approaches to simulate random attacks on edges to determine how cost-efficiency behaves [13, 49]. The steps involved in the simulation were the following:

**Step-1.** For a given time point, develop a group averaged weighted undirected functional connectivity network (*N_avg*) using the weighted functional connectivity networks (see Section 2.6.2 for details) of the 14 TBI subjects. Please note, in a subject specific weighted functional connectivity network, weight of each edge represents absolute correlation coefficient value. Now make an inverse network (*N_avg_inverse*) by subtracting the weight of each edge from 1.01. That is, if the average weight of an edge is 1, then we make it 0.01 so that it represents a short distance (but not a 0 distance) between the two ROIs; edges that are disconnected in *N_avg* also remain disconnected in *N_avg_inverse*, and are represented with a weight of 0 in both the networks.
**Step-2.** Define an integer variable *X* and initialize it to 1.
**Step-3.** Randomly delete *X%* edges from *N_avg_inverse* to produce a new network configuration.
**Step-4.** Evaluate the overall network cost and global efficiency of this new network and store it in a file.
**Step-5.** Replace back the deleted edges in the network, *N_avg_inverse*.
**Step-6.** Repeat step-3 to step-5 50 times.
**Step-7.** If *X* is less than 99
       Increment *X* by 1 and go to step-3.
     Else
       Stop.

**Fig 3:**
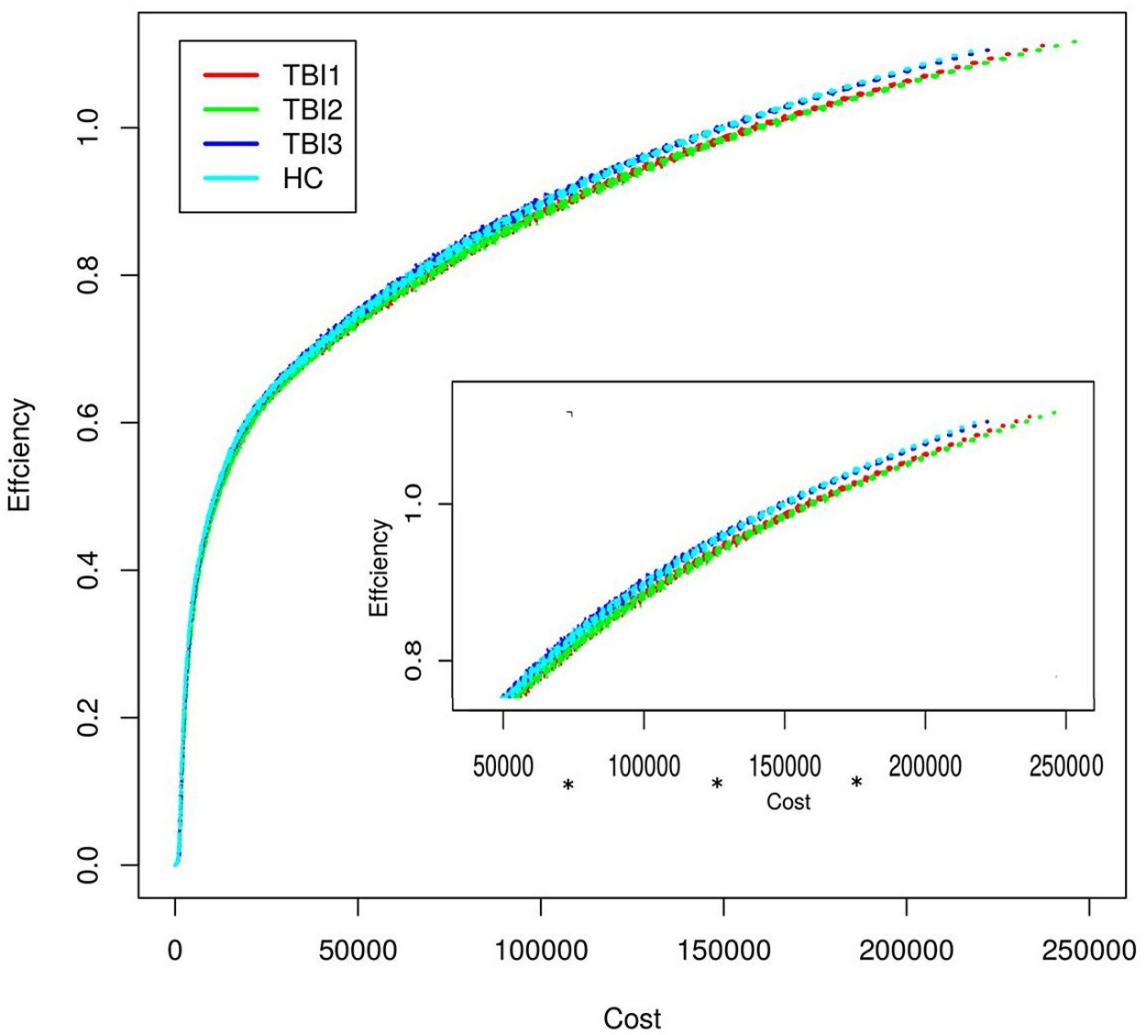
Illustrates cost-efficiency relationship of group-averaged TBI network at all 3 time points (see Section 3.1.4 for details). For cost bands 50000–100000, 100000–150000, and 150000–20000, there was a significant difference between the Time-2 and Time-3 efficiency values based on independent sample t-test. The Bonferroni corrected p-values and Cohen's-d for all cost bands are as follows, 0–50000 (p-value = 0.61, cohen's-d = 0.04), *50000–100000 (p-value << 0*.*05*, *cohen's-d = 0*.*30)*, *100000–150000 (p-value << 0*.*05*, *cohen's-d = 0*.*54)*, *150000–200000 (p-value << 0*.*05*, *cohen's-d = 0*.*70)*, and 200000–250000 (p-value = 1, cohen's-d = 0.06).

The output for the above procedure, at each time point is a single file (each file has 99x50 = 4950 rows) consisting of 2 columns: cost and efficiency. The cost and the efficiency values stored in these 3 files are then plotted as a graph (see Fig 3). We also ran the above simulation for healthy control subjects, to examine if the cost-efficiency relationship at Time-3 was similar to that of the healthy controls. Our results show, under random edge-attack, Time-3 is more robust and will maintain high efficiency for a much greater range of network densities (see cost of 50000 to 200000) than other time points. Also, the HC data are also plotted to provide context; Time-3 cost-efficiency relationship is more similar to the control subjects compared to this relationship at Time 2.

### 3.2 Local analysis

#### 3.2.1 Finding hyper/hypoconnected subsystems

**Fig 4:**
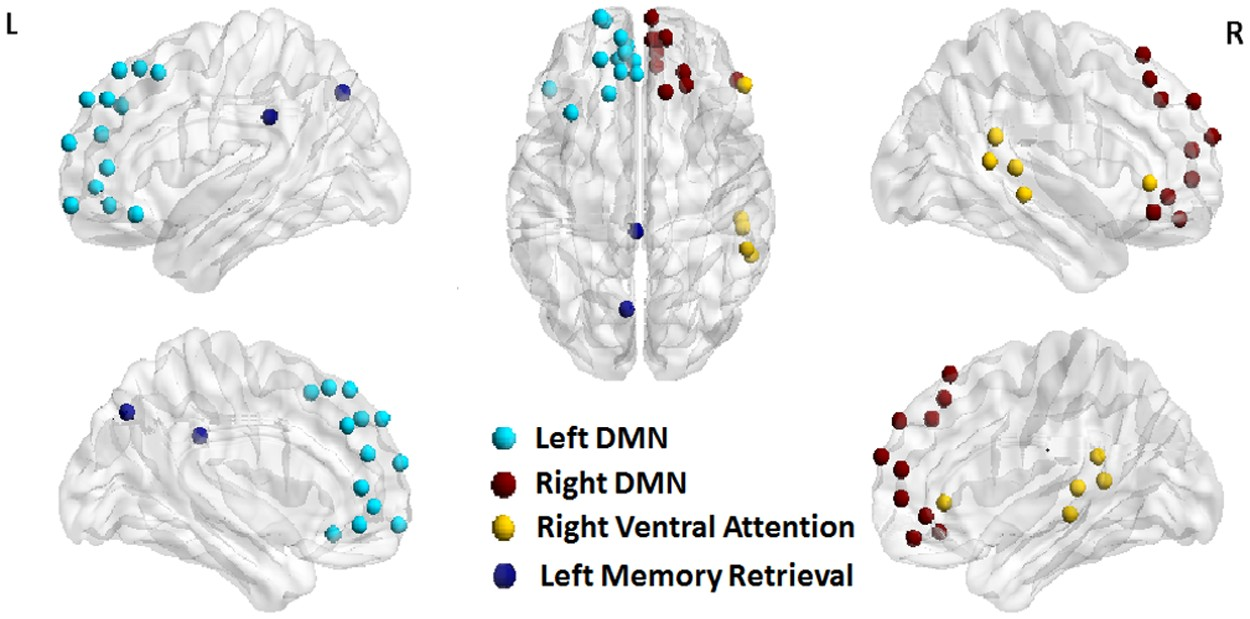
Hyper- and hypo-connected sub-systems in the brain found using local analysis (see Section 3.2.1 for details).

In order to isolate the most consistently observed regions showing hyper- and hypoconnectivity over the recovery period, we compared the TBI sample with the HC sample for network strength and cost as an initial “filter”. Global analysis demonstrated the most significant elevations in network strength and cost at Time-2, with no significant differences between the TBI and HC groups at Time points 1 and 3. Therefore, to reduce the number of analyses and to test specific hypotheses regarding hyperconnectivity in the brain’s subnetworks we grouped the 264 Power ROIs into 20 brain subsystems and compared Time-2 data (where hyperconnectivity was evident in global analysis; Table 5) to the HC data to determine regions of disproportionately greater connectivity. These 20 subnetworks derived from the Power 264 ROIs were based upon the brain’s most robust subnetworks and commonly sites of hyperconnectivity based upon the TBI literature (see S1 Table). For each brain subsystem, to examine hyperconnectivity we developed TBI and HC distributions of ROI-strengths (see section 2.6.3) using all ROIs that are part of the chosen subsystem. Then we performed independent sample t-tests comparing TBI and HC distributions. Thus, in all we performed 20 analyses for 20 collapsed brain systems for Time-2 data to direct further local analysis. The p-values were then corrected for multiple comparisons using FDR correction and in order to maintain sensitivity to medium effect sizes, we included result with a Cohen’s d > 0.70. If the mean of the TBI distribution was higher than the HCs it was labeled hyperconnected, and if it was less than the HCs then it was labeled hypo-connected. The results are summarized in Table 6 and the hyper- and hypo-connected sub-systems are shown in Fig 4.

**Table 6:** Hyperconnected and hypoconnected sub-systems for Time 2 TBI compared to HCs.

Sub-region description	Power Reg	P-value ^a^	Hyper/Hypo	Cohens-d
**Left Frontal DMN** ^‡^*	13	0.049	+	1.29
**Right Frontal DMN** ^‡^	10	0.051	+	1.14
Left Cingulate/Parietal Memory Retrieval*	2	0.179	-	-0.77
**Right Frontal Task Control**	9	0.051	+	1.11
**Right Parietal Task Control**	4	0.176	+	0.81
**Right Ventral Temporal/Parietal Attention**^‡^*	5	0.176	+	0.80

Power Reg, number of power regions in the sub-system; Hypo (-), hypoconnectivity; Hyper (+), hyperconnectivity; DMN, default mode network.

^a^ *p* values were False Discovery Rate (FDR) corrected.

^**‡**^ Significantly hyperconnected at Time 1.

* Significantly hyper/hypo connected at Time 3.

To examine cost, we performed an identical analysis as above for examining subsystem strength using findings from global Time-2 analysis to direct us to specific subsystems for within-group analysis. The results are summarized in Table 7. Based on the above strength and cost analyses for Time-2 data, several consistent regions demonstrated significantly higher strength and cost (Tables 6 and 7), so these regions served as the filter for examining Time-1-3 local effects.

**Table 7:** High cost brain sub-systems for Time 2 TBI compared to HC sample.

Sub-region description	Power Reg	P-value ^a^	Hyper/Hypo	Cohens-d
**Left Cingulate/Temporal DMN**	20	0.208	+	0.74
**Left Frontal DMN** *	13	0.037	+	**1.34**
**Right Frontal DMN** *	10	0.070	+	**1.08**
**Right Frontal Task Control**	9	0.070	+	1.06
**Right Parietal Task Control**	4	0.13	+	0.90
**Right Ventral Temporal/Parietal Attention**	5	0.208	+	0.71
**Right Dorsal Temporal/Parietal Dorsal Attention**	5	0.208	+	0.77

Power Reg, Number of power regions in the sub-system; Hypo (-), hypoconnectivity; Hyper (+), hyperconnectivity; DMN, default mode network.

^a^ *p* values were false discovery rate (FDR) corrected.

* Time 1 also significantly elevated for cost in these regions; No Time 3 also significantly elevated for cost in these regions.

Based upon the Time-2 filter, six regions were identified as high strength and seven regions as high cost for further analysis (Tables 6 and 7). To determine if these regions also showed significant increases at Time points 1 and 3, separate strength and cost analyses were conducted by comparing Time-1 and Time-3 data to the HC sample using an analysis of covariance with MRI scanner as a covariate in order to determine the influence of MRI scanner on results. Based upon a minimum of η_p_^2^ = 0.06 (small to medium effects size), at Time-1, three areas showed relatively greater strength in connections for the left DMN [F(1) = 2.4; p = 0.134; η_p_^2^ = 0.095], right DMN [F(1) = 2.6; p = 0.12; η_p_^2^ = 0.10], and the cingulo-parietal memory retrieval network [F(1) = 1.91; p = 0.179; η_p_^2^ = 0.07]. Time-1 also showed elevations in two subregions for cost: left DMN [F(1) = 2.3; p = 0.139; η_p_^2^ = 0.09] and right DMN [F(1) = 2.4; p = 0.13; η_p_^2^ = 0.10]. Similarly, for strength findings Time-1, the subregions Time-3 included hyperconnectivity for right frontal DMN and the right ventral parietal attention network and hypoconnectivity for the left cingulo-parietal memory retrieval network [left DMN: F(1) = 1.85; = 0.186; η_p_^2^ = 0.07; cingulo-parietal memory retrieval: F(1) = 5.7; p = 0.025, η_p_^2^ = 0.20; right ventral parietal attention: F(1) = 4.46; p = 0.046) η_p_^2^ = 0.16]. A primary difference at Time-3 compared to Time-1 and Time-2 was that no hyperconnected regions showed significantly elevated cost—even in left DMN and parietal attention networks. In a final analysis, we aimed to determine the difference in cost from Time-2 to Time-3 in these hyperconnected regions (below).

#### 3.2.2 Local cost x time analysis

**Fig 5:**
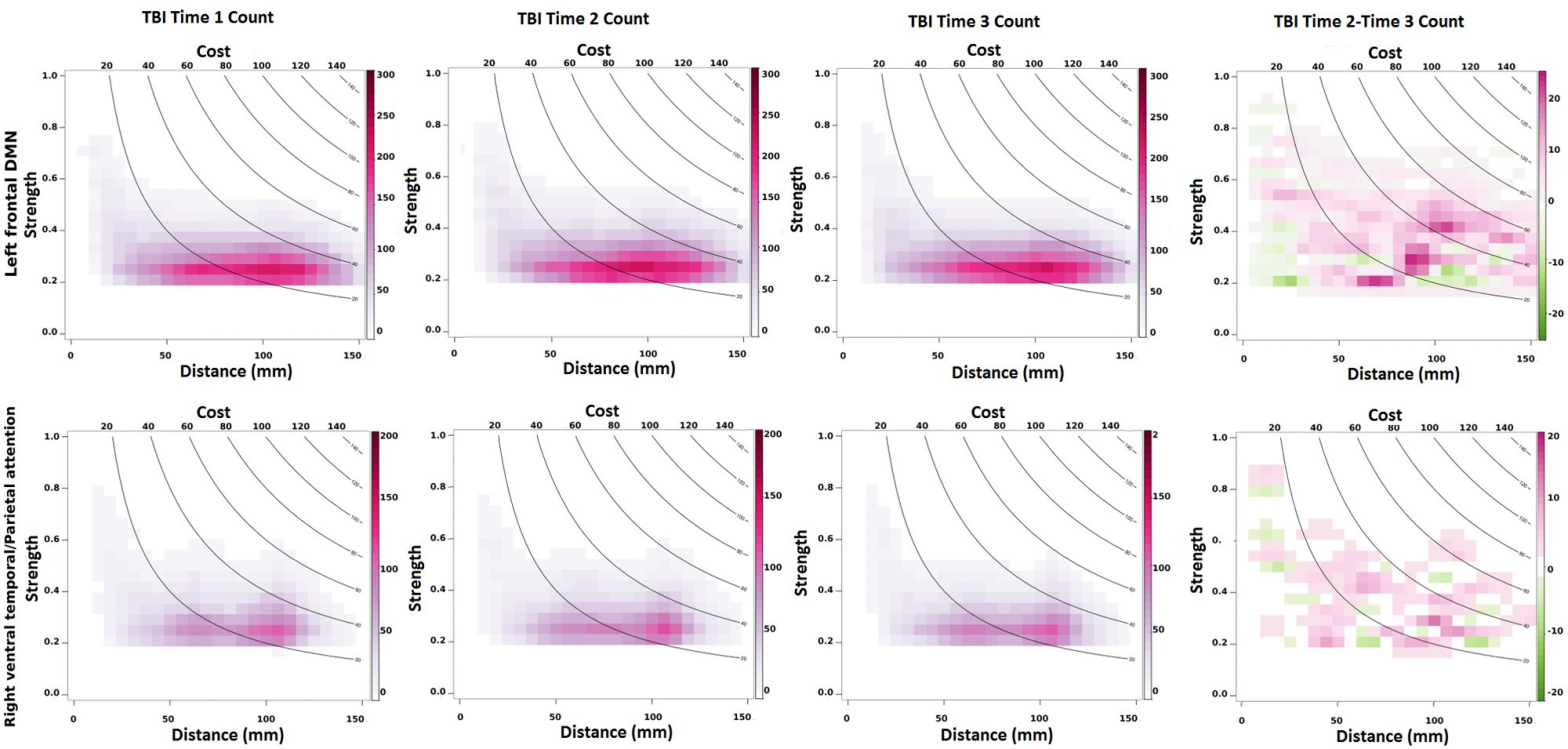
2-D histograms represent the distribution of connections that are incident upon ROIs within left frontal DMN (top) and Right Ventral Temporal/Parietal Attention (bottom) as a function of connection length and connection strength. A contour line (e.g. contour line of cost = 80) represents all possible values of connection distance and connection strength that can produce the same cost value (distance*strength).

Analyses comparing regions showing high connectivity and cost at Time-2 and Time-3 revealed hyperconnectivity in two subregions: the left frontal DMN and the ventral parietal attention network, but in the absence of increased cost at Time-3. To understand why these regions showed increased connectivity at both time points but heightened cost only at Time-2, we examined the factors that govern the cost (i.e. link strength, link distance, and link count). In this analysis, we first created a 2-dimensional (2-D) histogram of edge counts at each time point as follows (see Fig 5). For a given time point and for a given subsystem, for all connections that are incident upon the ROIs in the subsystem, the connection length and the connection strength of all TBI subjects were stored in a 2 column table. This table was then converted to a 2-D histogram where the color of each bin (or square box in the 2-D histogram) represents the number of connections that fall in that bin. These histogram plots were then overlaid with contour lines representing cost. A given contour line (e.g. contour line of cost = 80) represents all possible values of connection distance and strength that can produce the same cost value (distance*strength). For example, if the connection length is 40 mm and strength is 0.6 for a given connection, then the cost is 24, which is same as if the length was 60 mm and the strength was 0.4. Hence, both these points will fall on the same contour line, i.e. contour line of cost = 80. To compare Time-2 and Time-3 data, we then subtracted Time-3 histogram from Time-2 histogram to create a difference histogram. As shown in Fig 5, for Left Frontal DMN, Time-2 networks consisted of a greater number (pink regions) of medium/long length and medium/weak strength connections than Time-3 networks. A similar effect was also seen for Right Ventral Temporal/Parietal Attention regions; this effect appears to account for the cost differences in networks between Time-2 and Time-3 for these nodes.

#### 3.2.3 Behavioral analysis

To test whether the most consistently costly nodes within the network during recovery predict performance, we used bivariate Pearson correlations comparing cost and cognitive performance. To reduce the number of comparisons, we focused analyses on Time-2 and Time-3 connectivity data compared with three neuropsychological measures sensitive to deficits in TBI in the areas of executive control/switching (Trail Making Test), working memory (Digit Span Backward), and processing speed (VSAT total score). At 6 months post injury (Time-2), cost in the temporal-parietal attentional control region was significantly associated with performance on Digit Span backward (*r =* 0.777, *p =* 0.02); cost in this region was correlated with Trail Making Test (*r =* 0.462, *p =* 0.11), but showed no relationship with VSAT total (*r = -*0.052, *p =* 0.87). By contrasts, cost within the left frontal DMN showed little relationship with performance on Digits Backward (*r =* 0.033, *p =* 0.91) and Trail Making Test (*r =* 0.184, *p =* 0.55), but showed modest association with performance on the VSAT (*r =* -0.492, *p =* 0.09).

At 12 months post injury (Time-3), cost within the temporal-parietal attentional control region showed a modest association with performance on Digit Span Backward (*r =* 0.427, *p =* 0.15), and had little relationship with performance on Trail Making Test (*r =* -0.33, *p =* 0.92) and VSAT (*r =* 0.098, *p =* 0.75). Cost within the left frontal DMN showed the strongest association the Digit Span Backward (*r =* 0.664, *p =* 0.01), followed by performance on Trail Making Test (*r =* 0.410, *p =* 0.16) and the VSAT (*r =* -0.336, *p =* 0.26).

Overall, these data indicate that as cost increases, speed and executive control are reduced. Cost was positively associated with working memory performance, so there appears to be a trade-off between on-task processing efficiency and untimed working memory functioning. Indeed, in the TBI sample, working memory was negatively correlated with efficiency speeded performance.

#### 3.2.4: Supplementary brain parcellation using ICA

To examine the reliability of the findings in the global analysis, we introduced an identical connectivity analysis, changing only the method of brain parcellation. The initial findings using a network composed of Power 264 regions demonstrates high cost early after injury that partially resolves toward 1-year post injury. These findings are generally confirmed in the Supplementary analysis with one important difference: the timing of this resolution. The Supplementary analysis using ICA, high cost networks are more pronounced at Time 1 and this diminishes at Time 2 and Time 3 (see Tables A-C in S1 File). We offer an explanation for the differences in the timing of cost-efficiency resolution between the two approaches below.

Local analysis was highly consistent between the two separate analyses. The most highly connected node at all three time points is the anterior default mode network (medial left and right prefrontal cortex; see Figs A-C in S1 File) which overlaps with the primary finding from the Power 264 analysis in Table 7. Therefore, the locations for the expression of high cost (i.e., hubs) remain the same irrespective of analysis, but the global findings (e.g., network cost) are dependent upon the parcellation approach.

#### 3.2.5: Supplementary analysis: Influence of MRI scanner on connectivity results

In order to examine potential differences in tSNR between scanners, tSNR maps were generated for each visit of each subject based on the contrast between the mean signal of and the standard deviation of the EPI time-series for each voxel (after detrending with a second-order polynomial) (see Figs D-G in S1 File). For group analyses, the tSNR maps were normalized to the standard MNI brain space using the same normalization parameters for the fMRI data. The cross-system comparisons were conducted using one-way ANOVA with multiple comparisons. The longitudinal stability of tSNR for each MRI system was analyzed using the Sandwich Estimator for Neuroimaging Longitudinal Data (warwick.ac.uk/tenichols/SwE). There were no differences in tSNR in the same scanner across time, which is vital given the goals of this paper to examine the evolution of cost-efficiency during recovery. Between-scanner analyses revealed differences among the three scanners primarily in the mid-posterior cortical area of the inferior frontal lobe and the inferior cortical area of the temporal lobe. There are two important reasons why we do not anticipate that these differences between scanners influence the current findings. First, the higher tSNR occurs in the two scanners (Siemens HMC and Philips, Figs D-G in S1 File) where 8/12 HCs (and 21/26 total) were scanned, making tSNR as the determinant for hyperconnectivity in the TBI sample less intuitive. Second, there are few instances where tSNR overlaps spatially with the hubs that were seen as drivers of connectivity differences (e.g., insula; Figs F,G in S1 File). In summary, analysis of voxelwise tSNR revealed no observable influence of time on MRI signal which is vital to the central analyses presented in this paper. Additional, with regard to the few between-scanner findings observed, we anticipate that differences in scanner tSNR are not the source of the findings given the direction of these differences and the lack of spatial overlap with primary findings. We present these data in the Supplementary materials and make all data are publicly available.

## 4. Discussion

The current study used a graph theoretical analysis of whole-brain resting fMRI data and structural MRI to examine the changes in the brain connectivity during the first year following moderate and severe TBI. In doing so, this represents the first study to examine the evolution of network cost-efficiency trade-offs during recovery from neurological insult. Using a network of 264 functionally defined regions, hyperconnectivity was observable across distinct regions (e.g., hubs, peripheral nodes) and connection types (long-distance, short-distance connections) but, in the current data, increased connectivity peaked at about 6-months post injury (Time-2) before equilibrating in most subjects resulting in residual hyperconnectivity in only a few subnetworks by Time-3.

Hyperconnectivity at Time-3 did not result in significantly elevated cost and we interpret this to indicate that hyperconnectivity may be necessary to accommodate network disruption early after injury, but networks do not optimally sustain this early response. The relationship between early hyperconnectivity and later cost-efficiency trade-offs is elaborated upon in the following. Of note, there were important differences in network metrics based upon the parcellation approach used (Power 264 v ICA), but for reasons elaborated upon below, in this Discussion we focus our attention on the result of the analysis using the Power 264 parcellation approach.

### 4.1 Hyperconnectivity as a response to injury

The primary hypothesis that physical neurological disruption results in functional hyperconnectivity was supported; individuals with TBI showed increased network strength which peaked at around 6 months post injury (Time-2). Targeted analyses of the brain’s subnetworks revealed that the left frontal DMN and temporal-parietal attentional control regions were consistently hyperconnected at Time-1 and Time-2 and residual hyperconnectivity remained at Time-3, supporting hypothesis-2 that enhanced connectivity would be more evident in the “rich-club” regions (i.e. the most highly connected cortical hubs). Moreover, connectivity in frontal DMN and attentional control networks were both positively correlated with simple working memory rehearsal functioning at Time-3, but predicted reduced speed/efficiency, so the relationship between network cost and cognitive performance may not be entirely straight-forward.

Overall, the Time-3 findings presented here are largely consistent with the cross-sectional research examining TBI years post moderate and severe injury, showing residual hyperconnectivity in select subnetworks (see [3–7]). Given the significance of the injuries in this population, we endeavored to determine the influence of local pathology on large-scale networks using 3D lesion modeling. However, global metrics such as network strength and link count were not correlated with lesion percentage (total gray/lesion volume) and even exploratory analysis of the three regions significant connectivity differences at both Time-2 and Time-3 (e.g., left frontal DMN, cingulo-parietal memory network, parietal attention network) maintained only very small relationships to brain lesion percentage, with the highest at Time-3 ranging from r-values of 0.20 to 0.25.

In sum, hyperconnectivity was observed with significant elevations in connectivity at Time-2 and residual hyperconnectivity in network hubs at Time-3. The finding that Time-3 showed no regions of elevated cost paralleling the effects observed in Time-2 was an intriguing finding and became a focus of cost-efficiency analysis.

### 4.2 Balancing network cost-efficiency in the injured brain

The normally developed human cortex is organized so that local regions are more highly connected [13,17, 49–50] and connection density falls off as an inverse square function [51] which has been tied to optimal wiring to balancing cost-efficiency and may have important implications for how networks might adapt to injury. We aimed to examine the relationship between elevated connectivity and network cost after injury with particular interest in determining if hyperconnectivity occurred equally across distinct connection distances. To examine the relationship between hyperconnectivity and connection distance, we introduced novel metrics to estimate the cost of each functional connection by considering their length and the strength.

Examination of the regional differences in network cost revealed that bilateral frontal DMN maintained elevated cost at Time-1 and Time-2 (see Table 7). By contrast, the increased network cost observed at the first two time points diminished by Time-3 so that while subtle effects of hyperconnectivity were apparent at Time-3 it did not result in elevated network cost. In Fig 5, we examined the factors that govern the cost (i.e. link strength, link distance, and link count); a contour line represents all possible values of connection distance and strength that can produce the same cost value (distance*strength). These data reveal that the elevation in Time-2 cost is most likely attributable to a higher number of medium-range connections (as opposed to long-distance connections as anticipated). This finding is important as it could provide heuristics for understanding the edges most likely to be exploited during the expression of network plasticity and is likely related to the higher probability for observing hyperconnectivity in centrally located medial structures.

In a global analysis designed to examine cost-efficiency over time, simulations revealed that cost-efficiency was lowest at Time-2 (even less efficient than Time-1) and was optimized by Time-3 resulting in residual relative hyperconnectivity but diminished overall cost (Fig 3). We interpret this finding to indicate that Time-2 cost is not sustainable and the system adapts to a less costly, but efficient network. Several imaging studies have confirmed that the functional connectivity network of a healthy brain is configured to produce high efficiency (i.e. efficiency of information exchange) at a low connection cost [51–52]; i.e., healthy brains have been shown to exhibit some small-world-like characteristics. It is reasonable to assume that maintaining such high cost networks is accompanied by unwanted metabolic demand resulting in a network configuration that achieves higher efficiency at Time-3.

We observed elevated connectivity in the same sites at all three time points. Enhancing connectivity via hubs during recovery is consistent with the principle of preferential attachment [53,54], where the most highly connected nodes are the sites for integration of additional connections. These effects have been observed in animal models of neurodevelopment where the “rich get richer” during the formation of neural networks [55]. Hyperconnectivity via hubs after injury achieves two important goals. First, it may provide the opportunity to re-establish connectivity while maintaining an efficient network architecture. Second, centralized hub nodes are the most metabolically efficient regions in the brain [56]. Therefore, enhancing the connections in rich-club nodes permit re-establishing network functioning during critical recovery windows while balancing essential cost/efficiency trade-offs.

### 4.3 Influence of brain parcellation approach on findings

In a Supplementary Analysis, using a nearly identical processing stream up to the brain parcellation where we employed a spatially constrained ICA for comparison with the Power 264 functional atlas. Comparison of these two sets of results revealed nearly identical spatial overlap for regions with the highest cost in the TBI sample (e.g., medial frontal cortex, ECN). However, at the global level, there was partial resolution of hyperconnectivity by Time 2 as opposed to Time 3 observed in the Power 264 analysis. We anticipate that Time 2 hyperconnectivity was less evident using ICA because the most highly connected areas contributing to these effects using a Power 264 parcellation were collapsed into fewer nodes. Support for this interpretation is evident when inspecting the regional overlap between the two analyses. Unlike Time 1 and Time 3, for Time 2 all of the 10 most highly connected regions in the Power 264 analyses were contained within parts of the DMN and ECN. Mirroring this finding in Power 264, the three highest cost regions in the ICA at Time 2 included these same networks (see Figs A-C in S1 File). Thus network cost at Time 2 was differentially driven by nodes within the DMN and ECN, which were under-sampled during ICA. To underscore this point, right and left frontal DMN were the two most highly connected regions at Time 2 comprising 23 distinct Power “DMN” nodes in the prefrontal cortex (see Table 6). By contrast, of the 46 total components used for the network analysis after the ICA sort, only 3 frontal DMN regions were retained for network analysis thus greatly reducing the number of possible connections and the associated network cost observed in the DMN at Time 2. We therefore attribute the differences in Time 2 global results to the detailed sampling represented in the Power 264 compared to the ICA. Given the spatial overlap of the local findings and the more detailed spatial sampling provided by the Power 264 within identical regions, we anticipate that our focus on the Power 264 data offers a nuanced network analysis of cost-efficiency.

### 4.3 The implications of hyperconnectivity and increased network cost

There is a 150-year history in the neurological sciences of “lesion mapping” with the goal of pairing specific injury sites to functional loss [57] and in TBI this has been most recently observed in the resting state functional connectivity literature by examining specific connection loss in association with identified white matter disruption (see [4,6]). Given this context, the somewhat paradoxical, but consistent, finding that functional hyperconnectivity can occur in conjunction with loss of structural integrity of the brain must be integrated into models of functional plasticity. Hyperconnectivity after TBI appears to occur independent of the form or severity of pathological insult and this is particularly true when pathology does not target network hubs; in fact there are common profiles of hyperconnectivity across distinct clinical samples [58]. This conceptualization is consistent with a recent review of the influence of lesions on neural networks [59] and, in the current study global connectivity metrics such as network strength and path length maintained very little relationship to overall lesion load using 3D lesion mapping (see Fig 1) and residual hyperconnectivity was observable in centralized hubs (e.g., left frontal DMN) at Time-3 even in the context of global white matter volume loss from Time-1 to Time-3 (measured via VBM, see Table 4). Therefore, we argue that in order to understand system-level plasticity after neurological disruption, it is essential to understand the active network *response* to injury as opposed to focusing on the primary *consequences* of injury, which has been the longstanding emphasis in lesion mapping.

The mechanisms responsible for hyperconnectivity after TBI remain unclear, but there is increasing evidence that injury results in enhanced network connectivity in subnetworks of the brain (see [58] review) and we aimed to quantify how this brain response might influence network cost during the first year after injury. Establishing the timeline for the expression of hyperconnectivity and increased network cost may have important implications for brain health over the life span. For example, given the growing evidence in both animal [60–62] and human [63–65] research that local hypermetabolism may be a predictor of pathological protein deposition (e.g., beta amyloid), determining the permanence of the expression of high cost hyperconnectivity after injury may hold important implications for pathological aging and long-term outcome after TBI [66–68].

It should be made clear that our definition of cost is based upon a measure that integrates the number, strength, and Euclidean (physical) distance for connections of each node. Euclidean distance represents a linear physical distance that does not conform to the natural shape of the white matter which is an important simplification for interhemispheric or u-shaped fibers. Moreover, signaling time is not constant with axonal diameter playing an important role in propagation times [69]. So while a measure of physical distance may not be sensitive to subtle cost dimensions in processing time and metabolism, we anticipate that the cost metric we use here is a good starting-point for a within-subject cost-efficiency analysis where these factors remain constant over time.

### 4.4. Study conclusions and limitations

The current approach provides an important opportunity to examine whole-brain connectivity over multiple observation points during a critical window of recovery. The current sample is unique to the TBI functional brain imaging literature with respect to injury severity and time line for assessment of network functioning. Moreover, TBI samples have commonly been heterogeneous with respect to age, ranging from late teens to mid-50s [4–7, 9, 10, 24,70] and time-post-injury often ranges from months to years even within the same sample [4,7,9,70]. Both of these factors have influence on the observed network response and have therefore been addressed with this relatively homogeneous sample with respect to age. Also, most studies to date have focused on a limited number of regions-of-interest (ROIs) [3–5,9–10,70], or relied-upon seed-based (10) or averaging signal across large ROIs determined via anatomical atlases [25] leaving unknown the effects of TBI on more finely parcellated networks. Even so, this study is not without limitations requiring discussion.

First, much work remains to be done in order to determine those factors that define the balance of hyper/hypo connectivity in distributed networks, the primary neural substrates involved and their role in cognitive performance, and the temporal dynamics of these subnetworks when examined over longer windows of time (e.g. establishing the stationarity of connections). If windows of network plasticity can be established, it may serve as the foundation for more focused research on: 1) healthy and pathological mechanisms of network change and 2) stage-specific interventions during periods of both recovery and decline.

Because this study required data collection over a long window of time (~7 years) and at separate sites, it required the inclusion of multiple MRI scanners during data acquisition. Between-site data collection of MRI data remains an important methodological challenge for longitudinal work and multi-center studies [71–72] and we made efforts to guarantee the comparability of the signal-noise level across systems and the stability of each system over the study period with routine quality assurance scanning and analysis. In all formal between-group analysis of the data we included “MRI scanner” as a covariate to determine the contribution of scanner. Moreover, we conducted whole-brain voxelwise analysis of the tSNR revealing no differences in SNR in the same scanner over time which is vital to the within-subject hypotheses presented here. Between-scanner analysis revealed only subtle differences between the Philips and Siemens machines with each showing greater SNR in some locations (see Figs D,F in S1 File). More significant differences were evident when comparing the two Siemens scanners (see Fig E in S1 File), but for reasons discussed above, it is not clear how these regional affects would account for the current findings given the lack of spatial overlap. We do anticipate that scanner affects make it more difficult to observe consistent results between-groups and over time so we may lack sensitivity to very subtle effects during network analysis so the data collection across scanners remains a limitation of this study. The resting fMRI data included ample signal for examining connectivity (145 volumes), but remains only a brief representation of functioning in a dynamic system. Also, there are difficulties endemic to TBI when engaging in long, continuous scanning (e.g., fatigue, head movement), but longer time series provide the opportunity for examining the reliability of the results. While this is the largest sample of its kind in a study of recovery in moderate to severe TBI, the sample here remains small by behavioral study standards and a larger sample would permit more nuanced investigation of the relationship between distinct injury and clinical profiles and brain connectivity.

Finally, to test the reliability of these findings, we provide examination of distinct data parcellation techniques (e.g., Power 264 v. ICA) revealing an influence of parcellation approach on global metrics, but not in local findings. The influence of brain parcellation on network metrics is not surprising given the differences in the number of nodes (264 vs. 46) and the nature of the signal (i.e., averaged signal over a 15 mm cube of voxels vs. statistically derived “component”). The juxtaposition of the data resulting from these approaches is confirmatory regarding the specific regions showing the greatest contribution to elevated network cost after injury, but are a reminder that global metrics are node and degree dependent (see [66]).

## Supporting information

S1 Table List of 20 brain sub-systems. (DOCX)

S1 File Tables A-C: Network hubs for ICA-based network analysis for Times 1–3. Regions highly overlapping between time points and with the primary Power-264 parcellation analysis. Figs A-C: Global and local cost findings for ICA-derived network. Legend: IC = independent component from GIFT, PFC = prefrontal cortex, DMN = default mode network, ECN = executive control network, HF = high frequency, LF = low frequency. Note: “network cost” is for all edges to all nodes surviving correct p<0.05, FDR correction. Figs D-G: All analyses corrected Using FWE at p<0.05. MRI machines are: 1) Philips Achieva 3T MRI, 2) Siemens Magentom Trio 3T at Hershey Medical Center, and 3) Siemens Magnetom Trio 3T, University Park. Scanners 1 and 2 showed the greatest SNR. Regions of increased tSNR between HMC Siemen’s and Philips Machines.
(PPTX)
