# Correction: The evolution of cost-efficiency in neural networks during recovery from traumatic brain injury

**DOI:** 10.1371/journal.pone.0206005

**Published:** 2018-10-12

**Authors:** Arnab Roy, Rachel A. Bernier, Jianli Wang, Monica Benson, Jerry J. French, David C. Good, Frank G. Hillary

The Data Availability statement for this paper is incorrect. The correct statement is: Data are available from Openfmri at: ds000220.
