# Supplementary material for: The evolution of cost-efficiency in neural networks during recovery from traumatic brain injury
Source: PLoS One. 2017 Apr 19;12(4):e0170541. doi: 10.1371/journal.pone.0170541 (PMC5396850; doi:10.1371/journal.pone.0170541)
Supplement: S1 Table — (DOCX) [file pone.0170541.s001.docx]

**S1 Table: List of 20 brain sub-systems**

| **Sub-system** | **Number of ROIs in the Sub-system** |
| --- | --- |
| Left Frontal/Temporal Task Control | 7 |
| Right Frontal/Temporal Task Control | 7 |
| Left Cingulate/Temporal DMN | 20 |
| Right Temporal/Parietal DMN | 15 |
| Left Frontal DMN | 13 |
| Right Frontal DMN | 10 |
| Left Cingulate/Parietal Memory Retrieval | 2 |
| Right Cingulate/Parietal Memory Retrieval | 3 |
| Temporal/Parietal Visual | 4 |
| Right Frontal Task Control | 9 |
| Left Frontal Task Control | 9 |
| Left Frontal Task Control | 3 |
| Right Parietal Task Control | 4 |
| Right Frontal/Parietal Salience | 13 |
| Left Frontal/Cingulate DMN | 5 |
| Left Ventral Frontal/Temporal Attention | 4 |
| Right Ventral Temporal/Parietal Attention | 5 |
| Left Dorsal Temporal/Parietal Attention | 6 |
| Right Dorsal Temporal/Parietal Dorsal Attention | 5 |
| Cerebellum | 4 |
