# Supplementary material for: The evolution of cost-efficiency in neural networks during recovery from traumatic brain injury
Source: PLoS One. 2017 Apr 19;12(4):e0170541. doi: 10.1371/journal.pone.0170541 (PMC5396850; doi:10.1371/journal.pone.0170541)
Supplement: S1 File — Regions highly overlapping between time points and with the primary Power-264 parcellation analysis. Figs A-C: Global and local cost findings for ICA-derived network. Legend: IC = independent component from GIFT, PFC = prefrontal cortex, DMN = default mode network, ECN = executive control network, HF = high frequency, LF = low frequency. Note: “network cost” is for all edges to all nodes surviving correct p<0.05, FDR correction. Figs D-G: All analyses corrected Using FWE at p<0.05. MRI machines are: 1) Philips Achieva 3T MRI, 2) Siemens Magentom Trio 3T at Hershey Medical Center, and 3) Siemens Magnetom Trio 3T, University Park. Scanners 1 and 2 showed the greatest SNR. Regions of increased tSNR between HMC Siemen’s and Philips Machines. (PPTX) [file pone.0170541.s002.pptx]

## Slide 1
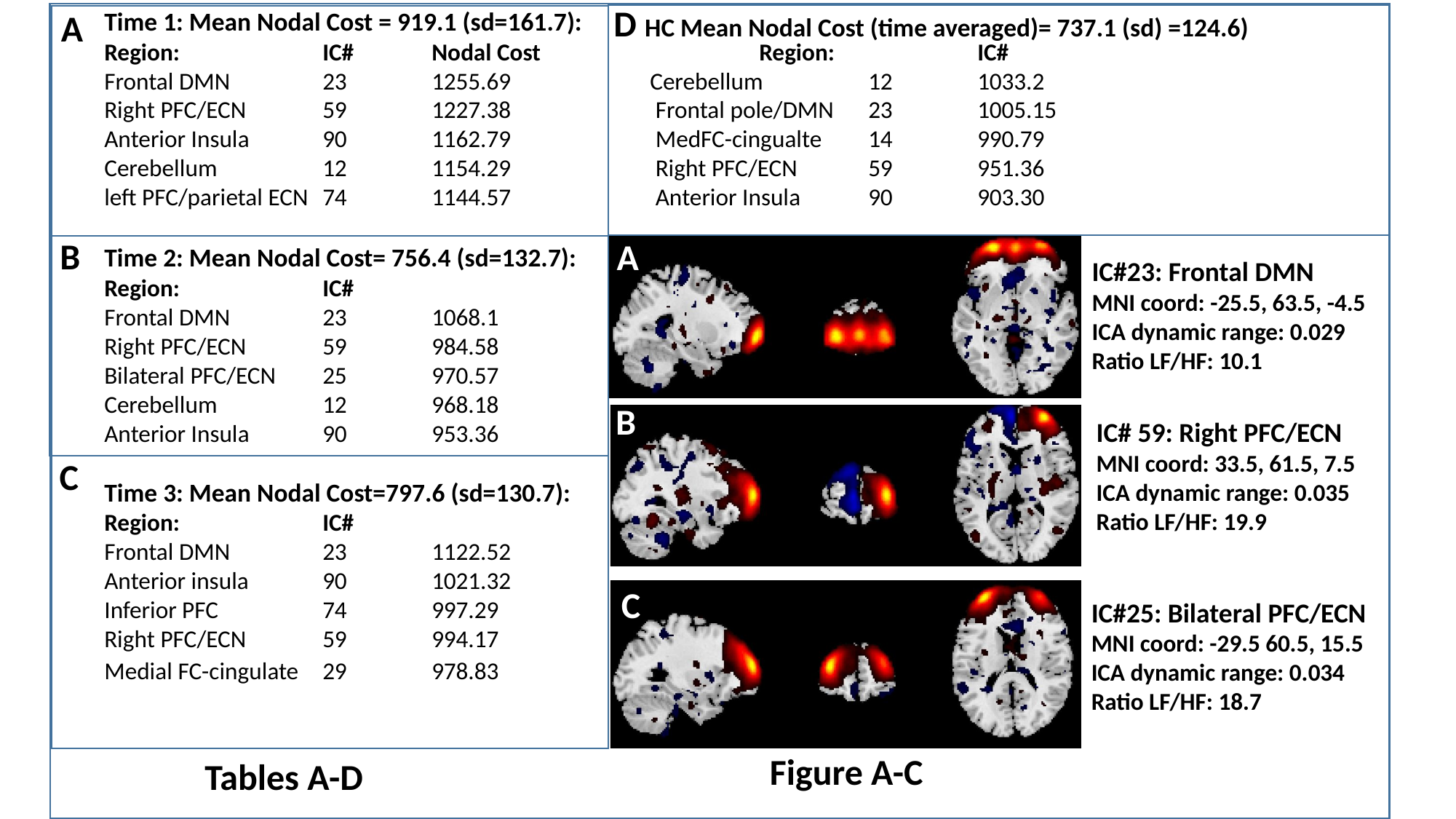

Time 1: Mean Nodal Cost = 919.1 (sd=161.7):
Region:		IC#	Nodal Cost		Region: 		IC#
Frontal DMN	23	1255.69		Cerebellum	12	1033.2
Right PFC/ECN 	59	1227.38		 Frontal pole/DMN	23	1005.15
Anterior Insula 	90	1162.79		 MedFC-cingualte	14	990.79
Cerebellum 	12	1154.29		 Right PFC/ECN	59	951.36
left PFC/parietal ECN	74	1144.57		 Anterior Insula 	90	903.30
Time 2: Mean Nodal Cost= 756.4 (sd=132.7):
Region:		IC#
Frontal DMN	23	1068.1
Right PFC/ECN 	59	984.58
Bilateral PFC/ECN	25	970.57
Cerebellum	12	968.18
Anterior Insula	90	953.36
Time 3: Mean Nodal Cost=797.6 (sd=130.7):
Region:		IC#
Frontal DMN	23	1122.52
Anterior insula	90	1021.32
Inferior PFC	74	997.29
Right PFC/ECN 	59	994.17
Medial FC-cingulate	29	978.83
D HC Mean Nodal Cost (time averaged)= 737.1 (sd) =124.6)
A
B
A
IC#23: Frontal DMN
MNI coord: -25.5, 63.5, -4.5
ICA dynamic range: 0.029
Ratio LF/HF: 10.1
B
IC# 59: Right PFC/ECN
MNI coord: 33.5, 61.5, 7.5
ICA dynamic range: 0.035
Ratio LF/HF: 19.9
C
C
IC#25: Bilateral PFC/ECN
MNI coord: -29.5 60.5, 15.5
ICA dynamic range: 0.034
Ratio LF/HF: 18.7
Figure A-C
Tables A-D

## Slide 2
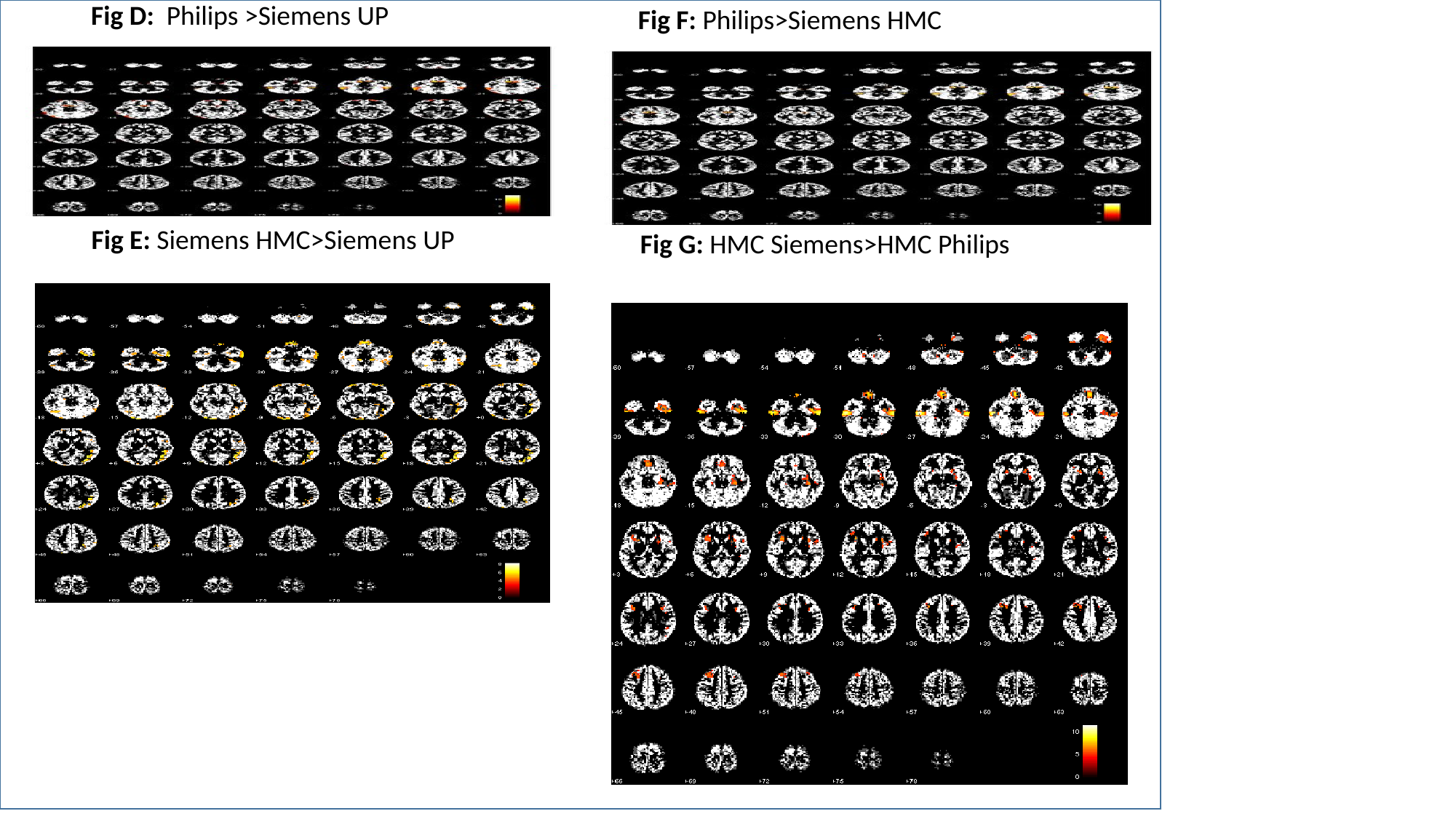

Fig D: Philips >Siemens UP
Fig F: Philips>Siemens HMC
Fig E: Siemens HMC>Siemens UP
Fig G: HMC Siemens>HMC Philips
